# Restoring Ovarian Fertility and Hormone Function: Recent Advancements, Ongoing Efforts and Future Applications

**DOI:** 10.1210/jendso/bvae073

**Published:** 2024-04-15

**Authors:** Elizabeth L Tsui, Hannah B McDowell, Monica M Laronda

**Affiliations:** Department of Pediatrics, Division of Endocrinology, Feinberg School of Medicine, Northwestern University, Chicago, IL 60611, USA; Stanley Manne Children's Research Institute, Ann & Robert H. Lurie Children's Hospital of Chicago, Chicago, IL 60611, USA; Department of Pediatrics, Division of Endocrinology, Feinberg School of Medicine, Northwestern University, Chicago, IL 60611, USA; Stanley Manne Children's Research Institute, Ann & Robert H. Lurie Children's Hospital of Chicago, Chicago, IL 60611, USA; Department of Pediatrics, Division of Endocrinology, Feinberg School of Medicine, Northwestern University, Chicago, IL 60611, USA; Stanley Manne Children's Research Institute, Ann & Robert H. Lurie Children's Hospital of Chicago, Chicago, IL 60611, USA; Department of Obstetrics and Gynecology, Feinberg School of Medicine, Northwestern University, Chicago, IL 60611, USA

**Keywords:** bioprosthetic ovary, fertility preservation, oncofertility, assisted reproduction technologies, ovarian tissue cryopreservation

## Abstract

The last 20 years have seen substantial improvements in fertility and hormone preservation and restoration technologies for a growing number of cancer survivors. However, further advancements are required to fill the gaps for those who cannot use current technologies or to improve the efficacy and longevity of current fertility and hormone restoration technologies. Ovarian tissue cryopreservation (OTC) followed by ovarian tissue transplantation (OTT) offers those unable to undergo ovarian stimulation for egg retrieval and cryopreservation an option that restores both fertility and hormone function. However, those with metastatic disease in their ovaries are unable to transplant this tissue. Therefore, new technologies to produce good-quality eggs and restore long-term cyclic ovarian function are being investigated and developed to expand options for a variety of patients. This mini-review describes current and near future technologies including in vitro maturation, in vitro follicle growth and maturation, bioprosthetic ovaries, and stem cell applications in fertility restoration research by their proximity to clinical application.

Advances in cancer therapies have led to substantial gains in survivorship [1]. In particular, the 5-year survival rate for children and adolescents with cancer has risen to above 85% [2]. Despite improved prognoses, life-saving treatments such as chemotherapy and radiation may result in premature gonadal insufficiency, or the inability to produce gametes and gonadal hormones [3]. Consequently, survivorship concerns such as fertility preservation and hormone restoration are increasingly prioritized by patients and physicians [4]. Not all cancer patients or patients who are undergoing chemotherapy or radiation treatments are at increased risk for loss of fertility and endocrine function. However, several medical societies, including those for pediatric patients, recommend that every patient with a new cancer diagnosis be counseled and educated on their risk [4-7]. Alkylating chemotherapies, radiation that targets the brain or abdomen, and stem cell conditioning regimens raises a patient's risk of gonadal insufficiency to “significant” or “high” [3]. Patients at high increased risk of developing premature gonadal insufficiency due to their treatments should be counseled on fertility preservation options.

Current options for fertility preservation are stratified by type of gonads present and pubertal status of the patient. Pubertal patients may use standard assisted reproductive technologies including gamete cryopreservation. However, for patients with ovaries, these techniques can be time intensive and require hormonal stimulation that is incompatible with the treatment plan. Furthermore, gamete cryopreservation is not feasible for prepubertal children who cannot undergo ovarian stimulation and egg retrieval. In contrast to pubertal individuals, whose oocytes are able to undergo meiotic maturation into fertilizable eggs, the immature hypothalamic-pituitary-gonadal axis in prepubertal individuals renders them insensitive to ovarian stimulation with oocytes that are not generally capable of normal fertilization [8].

As the production of euploid eggs occurs along a bell-shaped curve that peaks between ages 21 and 32 years, the quantity and quality of eggs retrieved from adolescent and young adult patients is an important consideration for the development of new and improved reproductive technologies for this clinical population [9]. An option available both to prepubertal and pubertal individuals is ovarian tissue cryopreservation (OTC). OTC involves isolation and cryopreservation of the cortical region of the ovary, which contains primordial follicles that constitute the ovarian reserve, for future autologous retransplantation (ovarian tissue transplantation, OTT) [10]. More than 360 OTTs have been reported, resulting in more than 140 reported live births after OTC [11-13]. However, although cumulatively 95% of participants who undergo OTT have a return to endocrine function post transplantation, the average duration of endocrine function is approximately 2 to 5 years [11, 14-16]. With an estimated life expectancy for a recent cohort of pediatric cancer survivors being between 55.9 and 58.1 years, patients will likely require multiple tissue transplants for long-term hormone restoration [17, 18]. Thus, if OTT is further refined, OTC and subsequent OTT have potential clinical utility that allows for more physiologic hormone restoration when compared to current hormone replacement therapies in addition to fertility preservation. Nevertheless, the safety and efficacy of OTC have generated interest in the expansion of clinical indications to include other contexts in which patients experience premature ovarian insufficiency such as Turner syndrome. Of the 174 ovaries that were cryopreserved using OTC for Turner syndrome patients, 62 (35.2%) of the ovaries contained follicles and thus may be viable for future OTT [19-26].

While OTC and OTT are important breakthroughs from the last 20 years, these techniques have additional limitations. For example, a recent publication of a cohort of pediatric patients who have undergone OTC at an academic medical center indicates that more than 20% received a diagnosis of leukemia or lymphoma [27]. In these patients, OTT may be contraindicated as in vivo xenograft studies using ovarian tissue showed migration of cells with leukemia-specific markers outside the transplanted tissue [28]. Additionally, those with known disease in their ovary are ineligible for transplantation of tissue in its native form due to the unknown risk of reseeding malignancy [2, 29-31]. Therefore, while OTC has enabled patients who could not undergo ovarian stimulation and oocyte retrieval an opportunity to preserve their fertility, additional innovations must be developed to improve the longevity and function of ovarian tissue after transplant, make OTT safe for all patients, and design new options for fertility and hormone restoration. As a result, work to provide expanded access and availability of fertility preservation technologies for oncofertility applications and beyond is an exciting avenue of current scientific research. This mini-review describes current and near future technologies in fertility restoration research by their proximity to clinical application (Fig. 1).

**Figure 1.**
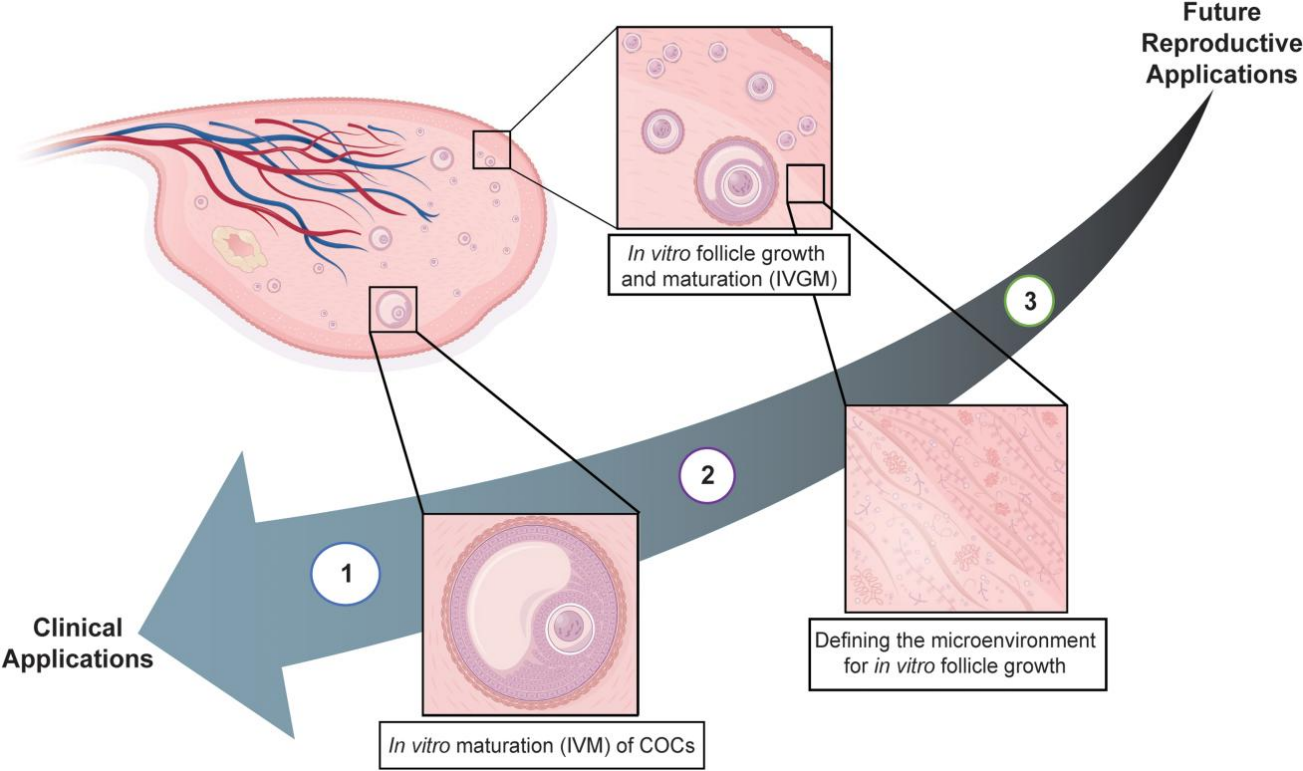
Fertility preservation and restoration advancements, from the bench to the clinic. Graphical representation of the advancements in fertility restoration from methodologies used currently in the clinic to early preclinical studies. (1) In the clinic: (egg retrieval and freezing; embryo preservation; OTC, ovarian tissue cryopreservation; and IVM, in vitro maturation of cumulus-oocyte-complexes [COCs] collected during OTC). (2) Rounding the mark: optimizing the culture and growth of immature eggs (IVGM, in vitro follicle growth with isolated follicles or in situ within tissue). (3) Ongoing preclinical work: defining the necessary microenvironment needed to engineer a bioprosthetic ovary for humans (ECM, extracellular matrix; soluble factors, paracrine signaling, physical cues, and vascularization).

## Ahead of the Pack: In Vitro Maturation

Closest to clinical application is an adaptation of current protocols used for in vitro fertilization (IVF), in vitro maturation (IVM) of cumulus-oocyte-complexes (COCs). COCs are aggregates of an oocyte surrounded by cumulus granulosa cells and can be retrieved from antral follicles following ovarian stimulation [32]. Often COCs collected with immature oocytes can undergo IVM to induce meiosis II in vitro, allowing for cryopreservation of mature, fertilizable eggs. COCs can also be released from antral follicles during ovarian tissue processing for OTC [33]. These recovered COCs may contain oocytes that are developed enough to undergo IVM. A recent systematic review of 12 studies where oocyte cryopreservation was performed with the COCs obtained during ovary tissue processing for OTC found that only 33% of recovered oocytes were able to be matured using current IVM technologies in a mixed population of pediatric and adult patients [34]. While there were some differences in the IVM protocols across centers, this is a reduced rate of maturation compared to adult-only IVF populations, where rates of mature metaphase II (MII) eggs resulting from IVM of recovered oocytes is approximately 70% [35]. These data indicate that age and pubertal status still significantly affect outcomes; however, clinicians and researchers have identified mature oocytes as well as oocytes that have successfully undergone IVM to produce developmentally competent eggs in the OTC processing media both from pediatric and adult patients [36-41]. Therefore, though pediatric patients may continue to face challenges, IVM provides an opportunity for pediatric oncofertility, where options are currently severely limited [37, 38, 42-45].

Many institutions continue to encourage egg cryopreservation over OTC because egg cryopreservation is a standard procedure for IVF clinics. However, oocytes from adolescents and young adults mature at a lower rate than their adult counterparts, and resulting eggs are less likely to be euploid [9, 46, 47]. This raises concerns regarding the future fertilization potential of eggs obtained from very young patients even when IVM is successful. An in vitro technique that can produce good-quality eggs from follicles of earlier stages (primordial, primary, or secondary) within ovarian tissue cryopreserved for future use may provide a controlled environment for growth and maturation and thus deliver a solution for these patient populations.

## Rounding the Corner: In Vitro Follicle Growth and Maturation

Techniques to recapitulate folliculogenesis, or follicle development, in vitro are collectively termed in vitro follicle growth and maturation (IVGM) and aim to generate well-developed follicles that can progress to IVM as described earlier. These technologies can be classified into follicle isolation and culture methodologies or in situ methods, where follicles remain embedded in the native ovarian tissue [48-57]. Irrespective of technique, follicle stage is critical to overall outcomes and stage-specific adaptations are required to optimize success rates. Primordial follicles are the initial, quiescent stage of follicles, the most abundant stage of oocytes that exist in prepubertal ovaries and are cryopreserved during OTC, making them a great source of potential eggs for patients. However, primordial follicles are incompletely surrounded by their supporting granulosa cells and are embedded within the dense, growth-prohibitive ovarian cortex. Together, these qualities make isolating intact primordial follicles challenging, requiring a series of mechanical and enzymatic steps [48-50, 58, 59]. This, alongside biologic variability, produces inconsistent results, ranging from 0 to more than 100 isolated follicles per participant [50, 59]. Recent studies in which primordial follicles were isolated and cultured in groups within hydrogel formulations (alginate, collagen, or fibrin) had mixed results regarding viability, with only an approximately 10% increase in follicle size during short-term culture [48, 50-53, 59]. Isolated primordial follicles may activate and begin to progress through folliculogenesis but cannot grow beyond primary follicles, the next major stage of folliculogenesis, without the presence of stromal cells [60]. In 1999, the first study to report the successful culture of primary follicles to early secondary-stage follicles was performed by encapsulating isolated follicles in collagen gels. These follicles, however, were short-lived and demonstrated only 40% viability after 24 hours [55]. Similar results were observed when primary follicles were cultured in alginate [56, 57]. The next follicle stage, secondary follicles, had improved viability in alginate, and a subset were able to develop into antral follicles after 30 days of culture [57]. Development to the antral stage is a key benchmark to allow for COC isolation and IVM, as discussed previously. To address limitations in the development of the earliest follicle stages, strategies have been developed to grow primordial follicles in situ to allow for improved isolation for IVGM.

The first report of human primordial follicles grown in situ, published in 1997, used thin sections of human ovarian cortex cultured on cell inserts and generated viable primary and secondary follicles following 21 days of culture [61]. The authors demonstrated that follicle viability was improved when tissue slices were cultured on an extracellular matrix (ECM)-rich substrate, Matrigel [61]. Further work optimizing the culture of human ovarian cortex slices has used defined culture media including alpha-minimum essential media, follicle-stimulating hormone, human serum albumin, and a mix of insulin-selenium-transferrin [62]. Additional studies in 2014 and 2015 demonstrated that ovarian cortical tissue pieces containing primordial and early transitional follicles can continue folliculogenesis into the antral stage when encapsulated in alginate hydrogels or when supported on ECM-rich “papers” [53, 63, 64]. Together, these results highlight the complexity of early follicle activation and growth in humans and a need for additional studies to generate a robust in vitro system of human follicle culture. Furthermore, these studies demonstrate the ability to identify and isolate viable ovarian follicles from human tissue in a variety of settings, with frozen-thawed tissue generally having no significant differences in follicle viability when compared to fresh tissue [45, 65-68].

Current challenges in IVGM include inefficient and unreliable growth and maturation of secondary follicles into eggs. Published protocols that have used secondary follicles isolated from human cortical tissue with or without in situ growth have resulted in 16 eggs of varying quality from 190 follicles from 84 patients [69-71]. These numbers are much lower than the 10 to 61 eggs required for a woman (aged 34-42 years) to have up to a 75% chance of conception with assisted reproductive technologies using high-quality sperm [72]. Given these limitations in current IVGM methods, a more efficient and reliable strategy to isolate, activate (primordial follicles), grow, and mature follicles to produce high-quality eggs is necessary to maximize fertility preservation options for patients. Effective IVGM for human primordial follicles and beyond likely requires a multistep, stage-specific, and complex culture system to support long-term growth and development into antral follicles that produce competent oocytes for IVM. Finally, while successful IVGM protocols would support the ability to have biological children, they would not address the restoration of ovarian hormone production. Therefore, additional technologies, including the bioprosthetic ovary discussed next, have been envisioned to couple long-term fertility and hormone restoration.

## Just off the Block: The Bioprosthetic Ovary—Proof of Concept in Murine Models

Advanced technologies, such as the bioprosthetic ovary, aim to restore complete ovarian function, extend the life of current ovarian tissue transplants, and advance fertility restoration options for individuals with disease in their cryopreserved ovarian tissue. Healthy follicles from a patient would be isolated and autologously transplanted into a defined, engineered environment—ensuring immune compatibility while recapitulating the functional components of the ovary. While currently undefined, considerable efforts are underway to identify optimal biomaterials for the bioprosthetic ovary that will support folliculogenesis and maturation in the clinic. In 2015, transplantation of recellularized ovarian scaffolds in ovariectomized mice allowed for estradiol (E_2_) production and follicle growth [73]. In 2017, full restoration of murine ovarian function was achieved when a bioprosthetic ovary, composed of donor follicles and a 3-dimensional–printed gelatin scaffold, was transplanted into an ovariectomized mouse [74]. The pups were born as a result of fertilization following natural ovulation of donor eggs from the bioprosthetic ovary after mating. However, additional research beyond this proof-of-concept study to determine how to increase transplant longevity and translate this bioprosthetic ovary to humans is required. One key condition that would influence the longevity of the transplant is the ability to regulate activation and growth of primordial follicles [75-78]. Recent studies suggest that primordial follicle activation is regulated at least in part by physical properties, and ovarian compartmentalization may provide clues as to how ovarian form supports function [58, 79].

## Just off the Block: The Bioprosthetic Ovary—Translational Efforts in Humans (Microenvironment)

Complicating translation of the aforementioned technology into humans is the complexity of the human ovarian microenvironment. The human ovary changes dynamically throughout fetal, postnatal, and pubertal development [79]. The fetal ovary is primarily composed of germ cells with little intervening interstitial cell populations [80]. However, at birth, approximately 40% of ovarian volume is composed of the interstitial component [80]. Pediatric ovaries undergo additional significant changes in size, shape, and subanatomic characteristics, completing compartmentalization into cortex and medulla at the same time that the individual demonstrates secondary sex characteristics [79]. These compartments contain unique interstitial populations, with adult ovaries demonstrating diverse cell populations including granulosa, theca/stroma, smooth muscle/perivascular, endothelial, and immune cells in addition to oocytes [81, 82]. Studies that track interstitial cell populations during murine ovary development identified a subset of cells that are recruited to form the steroid-producing theca layer of late-stage follicles, and functional in vitro studies have revealed that interstitial cell signaling is important for follicle activation, growth, and maturation [83-93]. Cyclical recruitment of ovarian follicles normally occurs under gonadotropin (follicle-stimulating hormone and luteinizing hormone)-dependent mechanisms established during puberty. Pediatric and pubertal patients make up a population that would substantially benefit from new restoration technologies and, as noted earlier, there are significant changes within the ovarian microenvironment in which follicles grow and oocytes mature at this time. Therefore, it is reasonable to conclude that the ovarian microenvironment that includes interstitial cell populations, neighboring follicles, secreted proteins, ECM proteins, and endocrine factors, can affect folliculogenesis and the development of a good-quality egg. Exciting work using in vitro systems to define these changes and their importance in follicle development is ongoing and is critical to determining optimal components of the human bioprosthetic ovary.

Standard in vitro assays used to test the effects of components on growth and maturation of isolated follicles use alginate hydrogel for its ease in gelation, biological inertness, and the ability to modify physical rigidity by toggling the alginate percentage or changing the calcium cross-linker [94]. However, alginate would not be useful in a bioprosthetic ovary intended to support long-term endocrine and ovulatory functions, as it is not easily remodeled by ovarian cells. Additionally, alginate does not support vessel infiltration, which limits key biological signaling that normally occurs through ECM interactions and reduces oxygenation and nutrient exchange of larger transplants.

In contrast to alginate, fibrin is readily degraded by proteolytic enzymes produced by granulosa and theca cells. In vitro, fibrin added to the alginate hydrogel (fibrin-alginate) enhanced the production of meiotically competent oocytes grown from secondary follicles and increased fertilization rates when compared to alginate alone [95, 96]. Fibrin-based materials have also been used to investigate follicle growth and survival in a variety of other species, including caprine, human, and rhesus macaque [97-99]. Isolated rhesus macaque follicles encapsulated in fibrin-alginate produced larger follicles, more E_2_, vascular endothelial growth factor (VEGF), and antimüllerian hormone when compared to alginate hydrogels alone [97]. These early studies showcase the ability to improve the production of meiotically competent oocytes in vitro for multiple species by altering the microenvironment of the system.

Further work to improve IVGM has identified other naturally occurring ovarian ECM proteins. For example, a comparison study of alginate and fibrin hydrogels found that murine follicles encapsulated in hyaluronic acid (HA)-alginate produced more eggs and significantly higher levels of E_2_ compared to alginate and fibrin hydrogels [100]. Desai et al [101] further supported this finding when murine follicles encapsulated in HA + Matrigel resulted in higher rates of germinal vesicle breakdown, MII formation, and higher E_2_ production when compared to controls. Ten years later, the authors reported functional competence when 82% of oocytes retrieved from follicles matured in HA hydrogels were capable of fertilization [102]. Because Matrigel comprises solubilized basement membrane derived from Engelbreth-Holm-Swarm mouse sarcoma making it rich in ECM and growth factors, the improved survival and oocyte quality in these studies can be explained by a variety of reasons [103]. As such, robust clinical application will require delineation of specific factors that contribute to increased follicle survival and oocyte quality.

To further understand the essential environmental cues that regulate folliculogenesis, a map of matrisome proteins and the physical properties of ovaries from model organisms and humans would be of great interest. The matrisome, which includes more than 1000 ECM and associated proteins, can contribute to folliculogenesis with both biochemical and biophysical cues [58, 104-106]. The physical environment is influenced by the density and composition of matrisome proteins. The ovarian cortex, which houses quiescent primordial follicles, is significantly more rigid than the medulla. Recently, a spatial map of porcine ovarian matrisome proteins was generated by proteomics analysis across 2 anatomical planes [107]. Out of the 82 matrisome proteins identified, 42 proteins were significantly differentially expressed across cortical and medullary compartments [107]. These differences in ovarian rigidity are important for folliculogenesis. In mice, treatment of the collagen-dense environment of the ovarian cortex with collagenase and trypsin increased primordial follicle activation in culture [58, 108]. The activation rate was restored by applying hyperbaric pressure, indicating that physical cues can maintain primordial follicle quiescence [58]. However, the reproductive biology field has yet to elucidate whether releasing primordial follicles from their environment alone can increase follicle activation through changes in mechanotransductive cues or whether these properties can be used to control activation [104, 109]. In addition to the physical properties of the matrisome, many proteins contain binding sites or act as ligands that influence transcriptional signals within neighboring cells [105]. Investigating these biochemical properties will be essential for designing a bioengineered environment for use in vitro or in vivo to improve fertility restoration options for patients.

## Just off the Block: The Bioprosthetic Ovary—Translational Efforts in Humans (Vascularization)

Vascular networks are essential for follicle growth, nutrient exchange, and peptide hormone transport in vivo. As follicles mature, increasing levels of VEGF are expressed by granulosa and theca cells, stimulating an independent vascular network around the follicle to support its development [110-112]. Therefore, designing materials that promote angiogenesis is essential for the development of a bioprosthetic ovary. Many have reported follicle survival and successful restoration of fertility in vivo using aggregated murine follicles or ovarian grafts encapsulated in fibrin or HA-supported matrices with and without additional growth factors [95, 96, 113-118]. Additional studies of human ovarian tissue that were encapsulated in fibrin clots with the addition of VEGF and xenografted into mice found significantly more proliferating follicles compared to unencapsulated tissue. Vascular structures that penetrated the fibrin-encapsulated tissue expressed both mouse-specific platelet endothelial cell adhesion molecule and human-specific von Willebrand factor, indicating that fibrin encapsulation with VEGF increased revascularization from both donor and recipient endothelial cells, improving follicular growth [119]. Importantly, fibrin glue is already being implemented in some human OTT procedures [120]. In addition to ECM and growth factors, Manavella et al hypothesized that fibrin embedded with proangiogenic adipose tissue–derived stem cells would improve revascularization, limiting hypoxia and ischemic injury to the tissue on transplantation [113-115]. The addition of stem cells in transplants resulted in increased pO_2_, total vessel area, primordial follicle survival, and reduced TUNEL-positive follicles [95-98]. These observations, with specific attention to essential microenvironmental factors and revascularization of tissue, are integral to the eventual design of a bioengineered ovary for human ovarian tissue or follicle transplantations.

## At the Starting Line: Building Follicles From Scratch

The far future of fertility preservation and restoration may lie in the ability to generate functional ovarian follicles from patients’ own stem cells. Recent reports have described the generation of oocytes and granulosa-like cells to generate functional ovarian follicles from induced pluripotent stem cells (iPSCs) [121]. This work was further extended to the generation of functional oocytes from induced pluripotent stem cells (iPSCs) of XY mice [122]. Additionally, significant efforts are underway to develop hormone-producing granulosa-like cells from human iPSCs, to expand fertility and endocrine restoration options for patients [123]. Although this work is far from a clinical application, it may eventually provide additional fertility preservation and restoration options for a variety of individuals.

## Conclusion

With a growing population of patients with premature gonadal insufficiency, there is a need to develop additional options for ovarian fertility and hormone restoration [124, 125]. There have been large advancements in fertility preservation and restoration technologies over the last few decades that are relevant for the practicing clinician. OTC and subsequent OTT is a favorable approach to restore fertility and endocrine function in patients at increased risk for developing premature gonadal insufficiency. However, the effectiveness of these technologies for the pediatric, adolescent, and young adult population must be thoroughly tested, and additional research to support improvements in the functionality and longevity of the ovarian transplant is needed [126, 127]. An overarching goal in the field is to develop new technologies that support fertility and hormone restoration in patients that are unable to use current assisted reproductive technologies, such as those pediatric patients with diseased cells in their ovaries. Development of good-quality human eggs will depend on our understanding of what makes the ideal microenvironment for primordial follicle activation, follicle growth, and oocyte maturation. Elucidating the roles of subanatomical, cellular, extracellular, and secreted factor components of the ovary will improve our understanding of human reproduction and support new ways for restoring function across diverse patient populations.

## Data Availability

Data sharing is not applicable to this article as no data sets were generated or analyzed during the current study.

## Abbreviations

COC: cumulus-oocyte-complex
E_2_: estradiol
ECM: extracellular matrix
HA: hyaluronic acid
iPSCs: induced pluripotent stem cells
IVF: in vitro fertilization
IVGM: in vitro follicle growth and maturation
IVM: in vitro maturation
MII, metaphase II; OTC: ovarian tissue cryopreservation
OTT: ovarian tissue transplantation
VEGF: vascular endothelial growth factor

## References

[1] Siegel RL , Miller KD, Sandeep N, Jemal A. Cancer statistics, 2023. CA Cancer J Clin. 2023;73(1):17‐48. 36633525 10.3322/caac.21763

[2] Miller KD , Fidler-Benaoudia M, Keegan TH, Hipp HS, Jemal A, Siegel RL. Cancer statistics for adolescents and young adults, 2020. CA Cancer J Clin. 2020;70(6):443‐459. 32940362 10.3322/caac.21637

[3] Meacham LR , Burns K, Orwig KE, Levine J. Standardizing risk assessment for treatment-related gonadal insufficiency and infertility in childhood adolescent and young adult cancer: the pediatric initiative network risk stratification system. J Adolesc Young Adult Oncol. 2020;9(6):662‐666. 32456570 10.1089/jayao.2020.0012

[4] Oktay K , Harvey BE, Partridge AH, et al Fertility preservation in patients with cancer: ASCO clinical practice guideline update. J Clin Oncol. 2018;36(19):1994‐2001. 29620997 10.1200/JCO.2018.78.1914

[5] Mead G . The effects of cancer treatment on reproductive functions. Clin Med. 2007;7:544‐549. 10.7861/clinmedicine.7-6-544 PMC4954356 18193698

[6] Ethics Committee of the American Society for Reproductive Medicine . Fertility preservation and reproduction in cancer patients. Fertil Steril. 005;83(6):1622‐1628. 15950628 10.1016/j.fertnstert.2005.03.013

[7] American Academy of Pediatrics Section on Hematology/Oncology Children's Oncology Group . Long-term follow-up care for pediatric cancer survivors. Pediatrics. 2009;123(3):906‐915. 19255020 10.1542/peds.2008-3688 PMC2696806

[8] Duncan FE , Schindler K, Schultz RM, et al Unscrambling the oocyte and the egg: clarifying terminology of the female gamete in mammals. Mol Hum Reprod. 2020;26(11):797‐800. 33022047 10.1093/molehr/gaaa066 PMC7648930

[9] Gruhn JR , Zielinska AP, Shukla V, et al Chromosome errors in human eggs shape natural fertility over reproductive lifespan. Science. 2019;365(6460):1466‐1469. 31604276 10.1126/science.aav7321 PMC7212007

[10] Silber SJ , Derosa M, Goldsmith S, Fan Y, Castleman L, Melnick J. Cryopreservation and transplantation of ovarian tissue: results from one center in the USA. J Assist Reprod Genet. 2018;35(12):2205‐2213. 30255455 10.1007/s10815-018-1315-1 PMC6289920

[11] Shapira M , Dolmans M-M, Silber S, Meirow D. Evaluation of ovarian tissue transplantation: results from three clinical centers. Fertil Steril. 2020;114(2):388‐397. 32605799 10.1016/j.fertnstert.2020.03.037

[12] Gellert SE , Pors SE, Kristensen SG, Bay-Bjørn AM, Ernst E, Yding Andersen C. Transplantation of frozen-thawed ovarian tissue: an update on worldwide activity published in peer-reviewed papers and on the Danish cohort. J Assist Reprod Genet. 2018;35(4):561‐570. 29497953 10.1007/s10815-018-1144-2 PMC5949119

[13] Donnez J , Dolmans M-M. Fertility preservation in women. N Engl J Med. 2017;377(17):1657‐1665. 29069558 10.1056/NEJMra1614676

[14] Diaz AA , Kubo H, Handa N, Hanna M, Laronda MM. A systematic review of ovarian tissue transplantation outcomes by ovarian tissue processing size for cryopreservation. Front Endocrinol (Lausanne). 2022;13:918899. 35774145 10.3389/fendo.2022.918899 PMC9239173

[15] Corkum KS , Rhee DS, Wafford QE, et al Fertility and hormone preservation and restoration for female children and adolescents receiving gonadotoxic cancer treatments: a systematic review. J Pediatr Surg. 2019;54(11):2200‐2209. 30773394 10.1016/j.jpedsurg.2018.12.021

[16] Dolmans M-M , von Wolff M, Poirot C, et al Transplantation of cryopreserved ovarian tissue in a series of 285 women: a review of five leading European centers. Fertil Steril. 2021;115(5):1102‐1115. 33933173 10.1016/j.fertnstert.2021.03.008

[17] Yeh JM , Ward ZJ, Chaudhry A, et al Life expectancy of adult survivors of childhood cancer over 3 decades. JAMA Oncol. 2020;6(3):350‐357. 31895405 10.1001/jamaoncol.2019.5582 PMC6990848

[18] Jensen AK , Kristensen SG, MacKlon KT, et al Outcomes of transplantations of cryopreserved ovarian tissue to 41 women in Denmark. Hum Reprod. 2015;30(12):2838‐2845. 26443605 10.1093/humrep/dev230

[19] Cheng J , Ruan X, Du J, et al Ovarian tissue cryopreservation for a 3-year-old girl with Mosaic Turner syndrome in China: first case report and literature review. Front Endocrinol (Lausanne). 2022;13:959912. 36479213 10.3389/fendo.2022.959912 PMC9719925

[20] Hreinsson JG , Otala M, Fridström M, et al Follicles are found in the ovaries of adolescent girls with Turner's syndrome. J Clin Endocrinol Metab. 2002;87(8):3618‐3623. 12161485 10.1210/jcem.87.8.8753

[21] Birgit B , Julius H, Carsten R, et al Fertility preservation in girls with turner syndrome: prognostic signs of the presence of ovarian follicles. J Clin Endocrinol Metab. 2009;94(1):74‐80. 18957497 10.1210/jc.2008-0708

[22] Balen AH , Harris SE, Chambers EL, Picton HM. Conservation of fertility and oocyte genetics in a young woman with mosaic Turner syndrome. BJOG. 2010;117(2):238‐242. 20002399 10.1111/j.1471-0528.2009.02423.x

[23] Huang JYJ , Tulandi T, Holzer H, et al Cryopreservation of ovarian tissue and in vitro matured oocytes in a female with mosaic Turner syndrome: Case Report. Hum Reprod. 2008;23(2):336‐339. 18056118 10.1093/humrep/dem307

[24] Peek R , Schleedoorn M, Smeets D, et al Ovarian follicles of young patients with Turner's syndrome contain normal oocytes but monosomic 45,X granulosa cells. Hum Reprod. 2019;34(9):1686‐1696. 31398245 10.1093/humrep/dez135 PMC6736193

[25] Dunlop CE , Jack SA, Telfer EE, Zahra S, Anderson RA. Clinical pregnancy in Turner syndrome following re-implantation of cryopreserved ovarian cortex. J Assist Reprod Genet. 2023;40(10):2385‐2390. 37566317 10.1007/s10815-023-02905-w PMC10504145

[26] Nadesapillai S , van der Velden J, van der Coelen S, et al TurnerFertility trial: fertility preservation in young girls with turner syndrome by freezing ovarian cortex tissue—a prospective intervention study. Fertil Steril. 2023;120(5):1048‐1060. 37549836 10.1016/j.fertnstert.2023.08.004

[27] McElhinney KL , Kennedy T, Rowell EE, Laronda MM. A dozen years of ovarian tissue cryopreservation at a pediatric hospital: tracking program and patient metrics while adapting to increasing needs. F S Rep. Published online February 16, 2024. Doi: 10.1016/j.xfre.2024.02.009 PMC11228781 38983744

[28] Shapira M , Raanani H, Barshack I, et al First delivery in a leukemia survivor after transplantation of cryopreserved ovarian tissue, evaluated for leukemia cells contamination. Fertil Steril. 2018;109(1):48‐53. 29198847 10.1016/j.fertnstert.2017.09.001

[29] Dolmans M-M , Marinescu C, Saussoy P, Van Langendonckt A, Amorim C, Donnez J. Reimplantation of cryopreserved ovarian tissue from patients with acute lymphoblastic leukemia is potentially unsafe. Blood. 2010;116(16):2908‐2914. 20595517 10.1182/blood-2010-01-265751

[30] Rosendahl M , Greve T, Andersen CY. The safety of transplanting cryopreserved ovarian tissue in cancer patients: a review of the literature. J Assist Reprod Genet. 2013;30(1):11‐24. 23263841 10.1007/s10815-012-9912-x PMC3553351

[31] Bockstaele L , Tsepelidis S, Dechene J, Englert Y, Demeestere I. Safety of ovarian tissue autotransplantation for cancer patients. Obstet Gynecol Int. 2012;2012:495142. 22253631 10.1155/2012/495142 PMC3255286

[32] Xie J , Xu X, Liu S. Intercellular communication in the cumulus–oocyte complex during folliculogenesis: a review. Front Cell Dev Biol. 2023;11:1087612. 36743407 10.3389/fcell.2023.1087612 PMC9893509

[33] De Roo C , Tilleman K. In vitro maturation of oocytes retrieved from ovarian tissue: outcomes from current approaches and future perspectives. J Clin Med. 2021;10(20):4680. 34682803 10.3390/jcm10204680 PMC8540978

[34] Mohd Faizal A , Sugishita Y, Suzuki-Takahashi Y, et al Twenty-first century oocyte cryopreservation—in vitro maturation of immature oocytes from ovarian tissue cryopreservation in cancer patients: a systematic review. Women's Health. 2022;18:17455057221114269. 10.1177/17455057221114269 PMC9393350 35983837

[35] Sacha CR , Kaser DJ, Farland LV, Srouji S, Missmer SA, Racowsky C. The effect of short-term exposure of cumulus-oocyte complexes to in vitro maturation medium on yield of mature oocytes and usable embryos in stimulated cycles. J Assist Reprod Genet. 2018;35(5):841‐849. 29536383 10.1007/s10815-018-1155-z PMC5984893

[36] Hanfling SN , Parikh T, Mayhew A, et al Case report: two cases of mature oocytes found in prepubertal girls during ovarian tissue cryopreservation. F S Rep. 2021;2(3):296‐299. 34553154 10.1016/j.xfre.2021.03.007 PMC8441562

[37] Fouks Y , Hamilton E, Cohen Y, Hasson J, Kalma Y, Azem F. In-vitro maturation of oocytes recovered during cryopreservation of pre-pubertal girls undergoing fertility preservation. Reprod Biomed Online. 2020;41(5):869‐873. 32843309 10.1016/j.rbmo.2020.07.015

[38] Karavani G , Wasserzug-Pash P, Mordechai-Daniel T, Bauman D, Klutstein M, Imbar T. Age-dependent in vitro maturation efficacy of human oocytes—is there an optimal age? Front Cell Dev Biol. 2021;9:667682. 34222236 10.3389/fcell.2021.667682 PMC8250136

[39] Hourvitz A , Yerushalmi GM, Maman E, et al Combination of ovarian tissue harvesting and immature oocyte collection for fertility preservation increases preservation yield. Reprod Biomed Online. 2015;31(4):497‐505. 26278808 10.1016/j.rbmo.2015.06.025

[40] Amargant F , Zhou LT, Yuan Y, et al FGF2, LIF, and IGF1 (FLI) supplementation during human in vitro maturation enhances markers of gamete competence. Hum Reprod. 2023;38(10):1938‐1951. 37608600 10.1093/humrep/dead162

[41] Nogueira D , Fajau-Prevot C, Clouet M, Assouline P, Deslandres M, Montagut M. Outcomes of different in vitro maturation procedures for oocyte cryopreservation for fertility preservation and yet another live birth in a cancer patient. Life. 2023;13(6):1355. 37374137 10.3390/life13061355 PMC10301157

[42] Duncan FE . Egg quality during the pubertal transition—is youth all It's cracked up to be? Front Endocrinol (Lausanne). 2017;8:226. 28928717 10.3389/fendo.2017.00226 PMC5591325

[43] Hartman CG . On the relative sterility of the adolescent organism. Science (1979). 1931;74(1913):226‐227. 10.1126/science.74.1913.226 17834965

[44] Ashley-Montagu MF . Adolescent sterility in the human female. Hum Fertil. 1946;11(2):33‐41. 20992572

[45] Kristensen SG , Rasmussen A, Byskov AG, Andersen CY. Isolation of pre-antral follicles from human ovarian medulla tissue. Hum Reprod. 2011;26(1):157‐166. 21112953 10.1093/humrep/deq318

[46] Karavani G , Schachter-Safrai N, Revel A, Mordechai-Daniel T, Bauman D, Imbar T. In vitro maturation rates in young premenarche patients. Fertil Steril. 2019;112(2):315‐322. 31056316 10.1016/j.fertnstert.2019.03.026

[47] Franasiak JM , Forman EJ, Hong KH, et al The nature of aneuploidy with increasing age of the female partner: a review of 15,169 consecutive trophectoderm biopsies evaluated with comprehensive chromosomal screening. Fertil Steril. 2014;101(3):656‐663.e1. 24355045 10.1016/j.fertnstert.2013.11.004

[48] Chiti MC , Dolmans M-M, Hobeika M, Cernogoraz A, Donnez J, Amorim CA. A modified and tailored human follicle isolation procedure improves follicle recovery and survival. J Ovarian Res. 2017;10(1):71. 29061149 10.1186/s13048-017-0366-8 PMC5654051

[49] Hovatta O , Wright C, Krausz T, Hardy K, Winston RML. Human primordial, primary and secondary ovarian follicles in long-term culture: effect of partial isolation. Hum Reprod. 1999;14(10):2519‐2524. 10527981 10.1093/humrep/14.10.2519

[50] Vanacker J , Camboni A, Dath C, et al Enzymatic isolation of human primordial and primary ovarian follicles with Liberase DH: protocol for application in a clinical setting. Fertil Steril. 2011;96(2):379‐383.e3. 21719006 10.1016/j.fertnstert.2011.05.075

[51] Camboni A , Van Langendonckt A, Donnez J, Vanacker J, Dolmans MM, Amorim CA. Alginate beads as a tool to handle, cryopreserve and culture isolated human primordial/primary follicles. Cryobiology. 2013;67(1):64‐69. 23688636 10.1016/j.cryobiol.2013.05.002

[52] Amorim CA , Van Langendonckt A, David A, Dolmans M-M, Donnez J. Survival of human pre-antral follicles after cryopreservation of ovarian tissue, follicular isolation and in vitro culture in a calcium alginate matrix. Hum Reprod. 2009;24(1):92‐99. 18815120 10.1093/humrep/den343

[53] Laronda MM , Duncan FE, Hornick JE, et al Alginate encapsulation supports the growth and differentiation of human primordial follicles within ovarian cortical tissue. J Assist Reprod Genet. 2014;31(8):1013‐1028. 24845158 10.1007/s10815-014-0252-x PMC4130945

[54] Abir R , Moore PA, Franks S, Margara RA, Mobberley MA, Winston RML. Mechanical isolation and in vitro growth of preantral and small antral human follicles. Fertil Steril. 1997;68(4):682‐688. 9341611 10.1016/s0015-0282(97)00264-1

[55] Abir R , Roizman P, Fisch B, et al Pilot study of isolated early human follicles cultured in collagen gels for 24 hours. Hum Reprod. 1999;14(5):1299‐1301. 10325281 10.1093/humrep/14.5.1299

[56] Wang T-R , Yan J, Lu C-L, et al Human single follicle growth in vitro from cryopreserved ovarian tissue after slow freezing or vitrification. Hum Reprod. 2016;31(4):763‐773. 26851603 10.1093/humrep/dew005

[57] Yin H , Kristensen SG, Jiang H, Rasmussen A, Andersen CY. Survival and growth of isolated pre-antral follicles from human ovarian medulla tissue during long-term 3D culture. Hum Reprod. 2016;31(7):1531‐1539. 27112699 10.1093/humrep/dew049

[58] Nagamatsu G , Shimamoto S, Hamazaki N, Nishimura Y, Hayashi K. Mechanical stress accompanied with nuclear rotation is involved in the dormant state of mouse oocytes. Sci Adv. 2019;5(6):eaav9960. 31249869 10.1126/sciadv.aav9960 PMC6594774

[59] Dolmans M-M , Michaux N, Camboni A, et al Evaluation of Liberase, a purified enzyme blend, for the isolation of human primordial and primary ovarian follicles. Hum Reprod. 2006;21(2):413‐420. 16199426 10.1093/humrep/dei320

[60] Gargus ES , Woodruff TK. Chapter 30: Contributions of ovarian stromal cells to follicle culture. In Fertil Preservation: Principles and Practice. 2021:341‐354. Doi:10.1017/9781108784368.031

[61] Hovatta O , Silye R, Abir R, Krausz T, Winston RML. Extracellular matrix improves survival of both stored and fresh human primordial and primary ovarian follicles in long-term culture. Hum Reprod. 1997;12(5):1032‐1036. 9194661 10.1093/humrep/12.5.1032

[62] Wright CS , Hovatta O, Margara R, et al Effects of follicle-stimulating hormone and serum substitution on the in-vitro growth of human ovarian follicles. Hum Reprod. 1999;14(6):1555‐1562. 10357975 10.1093/humrep/14.6.1555

[63] Henning NFC , Jakus AE, Laronda MM. Building organs using tissue-specific microenvironments: perspectives from a bioprosthetic ovary. Trends Biotechnol. 2021;39(8):824‐837. 33593603 10.1016/j.tibtech.2021.01.008 PMC8967215

[64] Jakus AE , Laronda MM, Rashedi AS, et al “Tissue papers” from organ-specific decellularized extracellular matrices. Adv Funct Mater. 2017;27(34):1700992. 29104526 10.1002/adfm.201700992 PMC5665058

[65] Silber S , Kagawa N, Kuwayama M, Gosden R. Duration of fertility after fresh and frozen ovary transplantation. Fertil Steril. 2010;94(6):2191‐2196. 20171622 10.1016/j.fertnstert.2009.12.073

[66] Silber S , Pineda J, Lenahan K, Derosa M, Melnick J. Fresh and cryopreserved ovary transplantation and resting follicle recruitment. Reprod Biomed Online. 2015;30(6):643‐650. 25892498 10.1016/j.rbmo.2015.02.010

[67] Fan Y , Flanagan CL, Brunette MA, et al Fresh and cryopreserved ovarian tissue from deceased young donors yields viable follicles. F S Sci. 2021;2(3):248‐258. 35146457 10.1016/j.xfss.2021.06.003 PMC8823279

[68] Kristensen SG , Liu Q, Mamsen LS, et al A simple method to quantify follicle survival in cryopreserved human ovarian tissue. Hum Reprod. 2018;33(12):2276‐2284. 30358835 10.1093/humrep/dey318

[69] McLaughlin M , Albertini DF, Wallace WHB, Anderson RA, Telfer EE. Metaphase II oocytes from human unilaminar follicles grown in a multistep culture system. Mol Hum Reprod. 2018;24(3):135‐142. 29390119 10.1093/molehr/gay002

[70] Xiao S , Zhang J, Romero MM, Smith KN, Shea LD, Woodruff TK. In vitro follicle growth supports human oocyte meiotic maturation. Sci Rep. 2015;5:17323. 26612176 10.1038/srep17323 PMC4661442

[71] Xu F , Lawson MS, Bean Y, et al Matrix-free 3D culture supports human follicular development from the unilaminar to the antral stage in vitro yielding morphologically normal metaphase II oocytes. Hum Reprod. 2021;36(5):1326‐1338. 33681988 10.1093/humrep/deab003 PMC8600176

[72] Goldman RH , Racowsky C, Farland L V, Munné S, Ribustello L, Fox JH. Predicting the likelihood of live birth for elective oocyte cryopreservation: a counseling tool for physicians and patients. Hum Reprod. 2017;32(4):853‐859. 28166330 10.1093/humrep/dex008

[73] Laronda MM , Jakus AE, Whelan KA, Wertheim JA, Shah RN, Woodruff TK. Initiation of puberty in mice following decellularized ovary transplant. Biomaterials. 2015;50:20‐29. 25736492 10.1016/j.biomaterials.2015.01.051 PMC4350019

[74] Laronda MM , Rutz AL, Xiao S, et al A bioprosthetic ovary created using 3D printed microporous scaffolds restores ovarian function in sterilized mice. Nat Commun. 2017;8:15261. 28509899 10.1038/ncomms15261 PMC5440811

[75] McLaughlin EA , McIver SC. Awakening the oocyte: controlling primordial follicle development. Reproduction. 2009;137(1):1‐11. 18827065 10.1530/REP-08-0118

[76] Llarena N , Hine C. Reproductive longevity and aging: geroscience approaches to maintain long-term ovarian fitness. J Gerontol A Biol Sci Med Sci. 2021;76(9):1551‐1560. 32808646 10.1093/gerona/glaa204 PMC8361335

[77] Monniaux D , Clément F, Dalbiès-Tran R, et al The ovarian reserve of primordial follicles and the dynamic reserve of antral growing follicles: what is the link? Biol Reprod. 2014;90(4):85. 24599291 10.1095/biolreprod.113.117077

[78] Roness H , Gavish Z, Cohen Y, Meirow D. Ovarian follicle burnout: a universal phenomenon? Cell Cycle. 2013;12(20):3245‐3246. 24036538 10.4161/cc.26358 PMC3885633

[79] Tsui EL , Harris CJ, Rowell EE, Laronda MM. Human ovarian gross morphology and sub-anatomy across puberty: insights from tissue donated during fertility preservation. F S Rep. 2023;4(2):196‐205. 37398615 10.1016/j.xfre.2023.02.008 PMC10310944

[80] Sforza C , Ferrario VF, De Pol A, Marzona L, Forni M, Forabosco A. Morphometric study of the human ovary during compartmentalization. Anat Rec. 1993;236(4):626‐634. 8379587 10.1002/ar.1092360406

[81] Wagner M , Yoshihara M, Douagi I, et al Single-cell analysis of human ovarian cortex identifies distinct cell populations but no oogonial stem cells. Nat Commun. 2020;11(1):1147. 32123174 10.1038/s41467-020-14936-3 PMC7052271

[82] Fan X , Bialecka M, Moustakas I, et al Single-cell reconstruction of follicular remodeling in the human adult ovary. Nat Commun. 2019;10(1):3164. 31320652 10.1038/s41467-019-11036-9 PMC6639403

[83] Reeves G . Specific stroma in the Cortex and medulla of the ovary. Cell types and vascular supply in relation to follicular apparatus and ovulation. Obstet Gynecol Surv. 1972;27(3):179‐181. 4143757

[84] Liu C , Peng J, Matzuk MM, Yao HH-C. Lineage specification of ovarian theca cells requires multicellular interactions via oocyte and granulosa cells. Nat Commun. 2015;6:6934. 25917826 10.1038/ncomms7934 PMC4413950

[85] Almeida AP , Saraiva M, Alves Filho JG, et al Gene expression and immunolocalization of fibroblast growth factor 2 in the ovary and its effect on the in vitro culture of caprine preantral ovarian follicles. Reprod Domest Anim. 2012;47(1):20‐25. 21518029 10.1111/j.1439-0531.2011.01793.x

[86] Sarabadani M , Tavana S, Mirzaeian L, Fathi R. Co-culture with peritoneum mesothelial stem cells supports the in vitro growth of mouse ovarian follicles. J Biomed Mater Res A. 2021;109(12):2685‐2694. 34228401 10.1002/jbm.a.37260

[87] Malekshah AK , Heidari M, Parivar K, Azami NS. The effects of fibroblast co-culture and activin A on in vitro growth of mouse preantral follicles. Iran Biomed J. 2014;18(1):49‐54. 24375163 10.6091/ibj.1264.2013 PMC3892140

[88] Heidari M , Malekshah AK, Parivar K, Khanbabaei R, Rafiei A. Effect of fibroblast co-culture on in vitro maturation and fertilization of mouse preantral follicles. Int J Fertil Steril. 2011;5(1):1‐8. 24917917 PMC4040237

[89] Chang H-M , Qiao J, Leung PCK. Oocyte–somatic cell interactions in the human ovary—novel role of bone morphogenetic proteins and growth differentiation factors. Hum Reprod Update. 2016;23(1):1‐18. 27797914 10.1093/humupd/dmw039 PMC5155571

[90] Ackert CL , Gittens JEI, O’Brien MJ, Eppig JJ, Kidder GM. Intercellular communication via Connexin43 gap junctions is required for ovarian folliculogenesis in the mouse. Dev Biol. 2001;233(2):258‐270. 11336494 10.1006/dbio.2001.0216

[91] Li S-Y , Bhandary B, Gu X, DeFalco T. Perivascular cells support folliculogenesis in the developing ovary. Proc Natl Acad Sci U S A. 2022;119(41):e2213026119. 10.1073/pnas.2213026119 PMC9564831 36194632

[92] Kim C-H , Cheon Y-P, Lee Y-J, et al The effect of fibroblast co-culture on in vitro maturation of mouse preantral follicles. Dev Reprod. 2013;17(3):269‐274. 25949142 10.12717/DR.2013.17.3.269 PMC4282301

[93] Yang Y , Kanno C, Sakaguchi K, Katagiri S, Yanagawa Y, Nagano M. Theca cells can support bovine oocyte growth in vitro without the addition of steroid hormones. Theriogenology. 2020;142:41‐47. 31574399 10.1016/j.theriogenology.2019.09.037

[94] Vanacker J , Amorim CA. Alginate: a versatile biomaterial to encapsulate isolated ovarian follicles. Ann Biomed Eng. 2017;45(7):1633‐1649. 28247039 10.1007/s10439-017-1816-6

[95] Shikanov A , Xu M, Woodruff TK, Shea LD. Interpenetrating fibrin-alginate matrices for in vitro ovarian follicle development. Biomaterials. 2009;30(29):5476‐5485. 19616843 10.1016/j.biomaterials.2009.06.054 PMC2906124

[96] Shikanov A , Xu M, Woodruff TK, Shea LD. A method for ovarian follicle encapsulation and culture in a proteolytically degradable 3 dimensional system. J Vis Exp. 2011;49:2695. 10.3791/2695 PMC3197327 21445043

[97] Xu J , Lawson MS, Yeoman RR, et al Fibrin promotes development and function of macaque primary follicles during encapsulated three-dimensional culture. Hum Reprod. 2013;28(8):2187‐2200. 23608357 10.1093/humrep/det093 PMC3712659

[98] Brito IR , Silva GM, Sales AD, et al Fibrin-alginate hydrogel supports steroidogenesis, in vitro maturation of oocytes and parthenotes production from caprine preantral follicles cultured in group. Reprod Domest Anim. 2016;51(6):997‐1009. 27650787 10.1111/rda.12779

[99] Chiti MC , Dolmans MM, Mortiaux L, et al A novel fibrin-based artificial ovary prototype resembling human ovarian tissue in terms of architecture and rigidity. J Assist Reprod Genet. 2018;35(1):41‐48. 29236205 10.1007/s10815-017-1091-3 PMC5758477

[100] Jamalzaei P , Valojerdi MR, Montazeri L, Baharvand H. Applicability of hyaluronic acid-alginate hydrogel and ovarian cells for in vitro development of mouse preantral follicles. Cell J (Yakhteh). 2020;22(Suppl 1):49‐60. 10.22074/cellj.2020.6925 PMC7481901 32779433

[101] Desai N , Abdelhafez F, Calabro A, Falcone T. Three dimensional culture of fresh and vitrified mouse pre-antral follicles in a hyaluronan-based hydrogel: a preliminary investigation of a novel biomaterial for in vitro follicle maturation. Reprod Biol Endocrinol. 2012;10(1):29. 22513305 10.1186/1477-7827-10-29 PMC3474165

[102] Desai N , Spangler M, Nanavaty V, Gishto A, Brown A. New hyaluronan-based biomatrix for 3-D follicle culture yields functionally competent oocytes. Reprod Biol Endocrinol. 2022;20(1):148. 36217168 10.1186/s12958-022-01019-9 PMC9549656

[103] Passaniti A , Kleinman HK, Martin GR. Matrigel: history/background, uses, and future applications. J Cell Commun Signal. 2022;16(4):621‐626. 34463918 10.1007/s12079-021-00643-1 PMC9733768

[104] Kawamura K , Cheng Y, Suzuki N, et al Hippo signaling disruption and Akt stimulation of ovarian follicles for infertility treatment. Proc Natl Acad Sci U S A. 2013;110(43):17474‐17479. 24082083 10.1073/pnas.1312830110 PMC3808580

[105] Hynes RO , Naba A. Overview of the matrisome—an inventory of extracellular matrix constituents and functions. Cold Spring Harb Perspect Biol. 2012;4(1):a004903. 21937732 10.1101/cshperspect.a004903 PMC3249625

[106] Naba A , Clauser KR, Ding H, Whittaker CA, Carr SA, Hynes RO. The extracellular matrix: tools and insights for the “omics” era. Matrix Biol. 2016;49:10‐24. 26163349 10.1016/j.matbio.2015.06.003 PMC5013529

[107] Henning NF , LeDuc RD, Even KA, Laronda MM. Proteomic analyses of decellularized porcine ovaries identified new matrisome proteins and spatial differences across and within ovarian compartments. Sci Rep. 2019;9(1):20001. 31882863 10.1038/s41598-019-56454-3 PMC6934504

[108] Bochner F , Fellus-Alyagor L, Kalchenko V, Shinar S, Neeman M. A novel intravital imaging window for longitudinal microscopy of the mouse ovary. Sci Rep. 2015;5:12446. 26207832 10.1038/srep12446 PMC4513547

[109] Lunding SA , Pors SE, Kristensen SG, et al Biopsying, fragmentation and autotransplantation of fresh ovarian cortical tissue in infertile women with diminished ovarian reserve. Hum Reprod. 2019;34(10):1924‐1936. 31593582 10.1093/humrep/dez152

[110] Xu X , Mu L, Li L, et al Imaging and tracing the pattern of adult ovarian angiogenesis implies a strategy against female reproductive aging. Sci Adv. 2022;8(2):eabi8683. 35020427 10.1126/sciadv.abi8683 PMC8754302

[111] Bruno JB , Matos MHT, Chaves RN, et al Angiogenic factors and ovarian follicle development. Anim Reprod. 2018 6(2):371‐379.

[112] Robinson RS , Woad KJ, Hammond AJ, Laird M, Hunter MG, Mann GE. Angiogenesis and vascular function in the ovary. Reproduction. 2009;138(6):869‐881. 19786399 10.1530/REP-09-0283

[113] Manavella DD , Cacciottola L, Desmet CM, et al Adipose tissue-derived stem cells in a fibrin implant enhance neovascularization in a peritoneal grafting site: a potential way to improve ovarian tissue transplantation. Hum Reprod. 2018;33(2):270‐279. 29304240 10.1093/humrep/dex374

[114] Manavella DD , Cacciottola L, Payen VL, Amorim CA, Donnez J, Dolmans MM. Adipose tissue-derived stem cells boost vascularization in grafted ovarian tissue by growth factor secretion and differentiation into endothelial cell lineages. Mol Hum Reprod. 2019;25(4):184‐193. 30824937 10.1093/molehr/gaz008

[115] Manavella DD , Cacciottola L, Pommé S, et al Two-step transplantation with adipose tissue-derived stem cells increases follicle survival by enhancing vascularization in xenografted frozen-thawed human ovarian tissue. Hum Reprod. 2018;33(6):1107‐1116. 29635371 10.1093/humrep/dey080

[116] Cacciottola L , Nguyen TYT, Chiti MC, et al Long-term advantages of ovarian reserve maintenance and follicle development using adipose tissue-derived stem cells in ovarian tissue transplantation. J Clin Med. 2020;9(9):2980. 32942743 10.3390/jcm9092980 PMC7564479

[117] Tanaka A , Nakamura H, Tabata Y, Fujimori Y, Kumasawa K, Kimura T. Effect of sustained release of basic fibroblast growth factor using biodegradable gelatin hydrogels on frozen-thawed human ovarian tissue in a xenograft model. J Obstet Gynaecol Res. 2018;44(10):1947‐1955. 29998469 10.1111/jog.13726

[118] Tavana S , Azarnia M, Valojerdi MR, Shahverdi A. Hyaluronic acid-based hydrogel scaffold without angiogenic growth factors enhances ovarian tissue function after autotransplantation in rats. Biomed Mater. 2016;11(5):055006. 27710922 10.1088/1748-6041/11/5/055006

[119] Magen R , Shufaro Y, Daykan Y, et al Use of simvastatin, fibrin clots, and their combination to improve human ovarian tissue grafting for fertility restoration after anti-cancer therapy. Front Oncol. 2021;10:598026. 33552971 10.3389/fonc.2020.598026 PMC7862713

[120] Dolmans M-M , Manavella DD. Recent advances in fertility preservation. J Obstet Gynaecol Res. 2019;45(2):266‐279. 30246274 10.1111/jog.13818

[121] Hikabe O , Hamazaki N, Nagamatsu G, et al Reconstitution in vitro of the entire cycle of the mouse female germ line. Nature. 2016;539(7628):299‐303. 27750280 10.1038/nature20104

[122] Murakami K , Hamazaki N, Hamada N, et al Generation of functional oocytes from male mice in vitro. Nature. 2023;615(7954):900‐906. 36922585 10.1038/s41586-023-05834-x

[123] Smela MDP , Kramme CC, Fortuna PRJ, et al Directed differentiation of human iPSCs to functional ovarian granulosa-like cells via transcription factor overexpression. Elife. 2023;12:e83291. 36803359 10.7554/eLife.83291 PMC9943069

[124] Chon SJ , Umair Z, Yoon M-S. Premature ovarian insufficiency: past, present, and future. Front Cell Dev Biol. 2021;9:672890. 34041247 10.3389/fcell.2021.672890 PMC8141617

[125] Li M , Zhu Y, Wei J, Chen L, Chen S, Lai D. The global prevalence of premature ovarian insufficiency: a systematic review and meta-analysis. Climacteric. 2023;26(2):95‐102. 36519275 10.1080/13697137.2022.2153033

[126] Rowell E , Duncan F, Laronda M. ASRM removes the experimental label from Ovarian Tissue Cryopreservation (OTC): pediatric research must continue. *Fertility and Sterility*. Published March 26, 2020. Accessed December 16, 2023. https://www.fertstert.org/news-do/asrm-removes-experimental-label-ovarian-tissue-cryopreservation-otc-pediatric-research

[127] Nahata L , Woodruff TK, Quinn GP, et al Ovarian tissue cryopreservation as standard of care: what does this mean for pediatric populations? J Assist Reprod Genet. 2020;37(6):1323‐1326. 32390071 10.1007/s10815-020-01794-7 PMC7311630

